# Genome Sequences of 15 SARS-CoV-2 Sublineage B.1.617.2 Strains in Bangladesh

**DOI:** 10.1128/MRA.00560-21

**Published:** 2021-07-15

**Authors:** Mokibul Hassan Afrad, Manjur Hossain Khan, Sadia Isfat Ara Rahman, Omar Hamza Bin Manjur, Mohabbat Hossain, Ahmed Nawsher Alam, Farzana Islam Khan, Nawroz Afreen, Farsim Tarannum Haque, Nicholas R. Thomson, Tahmina Shirin, Firdausi Qadri

**Affiliations:** a International Centre for Diarrhoeal Disease Research, Bangladesh, Dhaka, Bangladesh; b Institute of Epidemiology, Disease Control, and Research, Dhaka, Bangladesh; c Institute for Developing Science and Health Initiatives, Dhaka, Bangladesh; d Wellcome Sanger Institute, Hinxton, Cambridgeshire, United Kingdom; e London School of Hygiene and Tropical Medicine, London, United Kingdom; KU Leuven

## Abstract

We report the coding-complete genome sequences of 15 severe acute respiratory syndrome coronavirus 2 (SARS-CoV-2) sublineage B.1.617.2 strains that were obtained from Bangladeshi individuals with a history of recent travel to India and from the Bangladeshi community. Genomic data were generated by Nanopore sequencing using the amplicon sequencing approach developed by the ARTIC Network.

## ANNOUNCEMENT

Bangladesh reported the first confirmed case of severe acute respiratory syndrome coronavirus 2 (SARS-CoV-2) (family *Coronaviridae*, genus *Betacoronavirus*) on 8 March 2020; as of 31 May 2021, there were 798,830 confirmed coronavirus disease 2019 (COVID-19) cases (1). Bangladesh has been observing a surge of confirmed SARS-CoV-2 cases, dominated by the SARS-CoV-2 beta variant B.1.351, since the third week of March 2021 (2). However, the rate of confirmed SARS-CoV-2 cases nationwide gradually decreased to less than 10% (SARS-CoV-2 test positivity rate) in mid-May 2021. Recently (third week of May 2021), there was a substantial increase in the COVID-19 detection rate in the bordering districts (Chapainawabgonj, Khulna, and Satkhira) in the northwestern part of Bangladesh. We investigated whether this sudden increase was due to SARS-CoV-2 variants that might have been imported into Bangladesh through population movement in the Bangladesh-India border regions.

We obtained the SARS-CoV-2-positive samples (*n* = 15) through the nationwide COVID-19 surveillance network maintained by the Institute of Epidemiology, Disease Control, and Research (IEDCR), a mandated institute for disease outbreak investigation and response in Bangladesh. This genomic surveillance is an ongoing activity that was approved by the institutional review board of the IEDCR (protocol IEDCR/IRB/2020/11). These 15 samples include samples from (i) randomly selected SARS-CoV-2-positive community patients (*n* = 7) with COVID-19 symptoms who were residing in the border district of Chapainawabgonj and (ii) Bangladeshi individuals (*n* = 8) with a history of recent travel to India who were found to be SARS-CoV-2 positive by reverse transcriptase (RT) PCR, irrespective of COVID-19 symptom appearance (3). The patients’ ages ranged from 13 to 52 years; four of the patients were asymptomatic, while others presented mild to moderate symptoms. Except for one individual, none had been vaccinated prior to infection. Viral RNA was extracted from nasopharyngeal swab samples using the QIAamp viral RNA minikit (Qiagen). Sequencing libraries were prepared using the multiplex PCR amplicon sequencing approach developed by the ARTIC Network (4, 5). Libraries were multiplexed and sequenced on a FLO-MIN106D (R9.5) flow cell for at least 12 h. Raw reads were base called and demultiplexed with MinKNOW v21.02.1. Processed reads were assembled using the ARTIC guppyplex script with Medaka v1.4 using the ARTIC EPI2ME v3.3.0 SARS-CoV-2 pipeline (FastQ quality control plus ARTIC plus NextClade) (https://artic.network/ncov-2019/ncov2019-bioinformatics-sop.html). In total, 4,324,431 reads were obtained (range, 79,314 to 734,341 reads per sample; average length, 507 bp).

**FIG 1:**
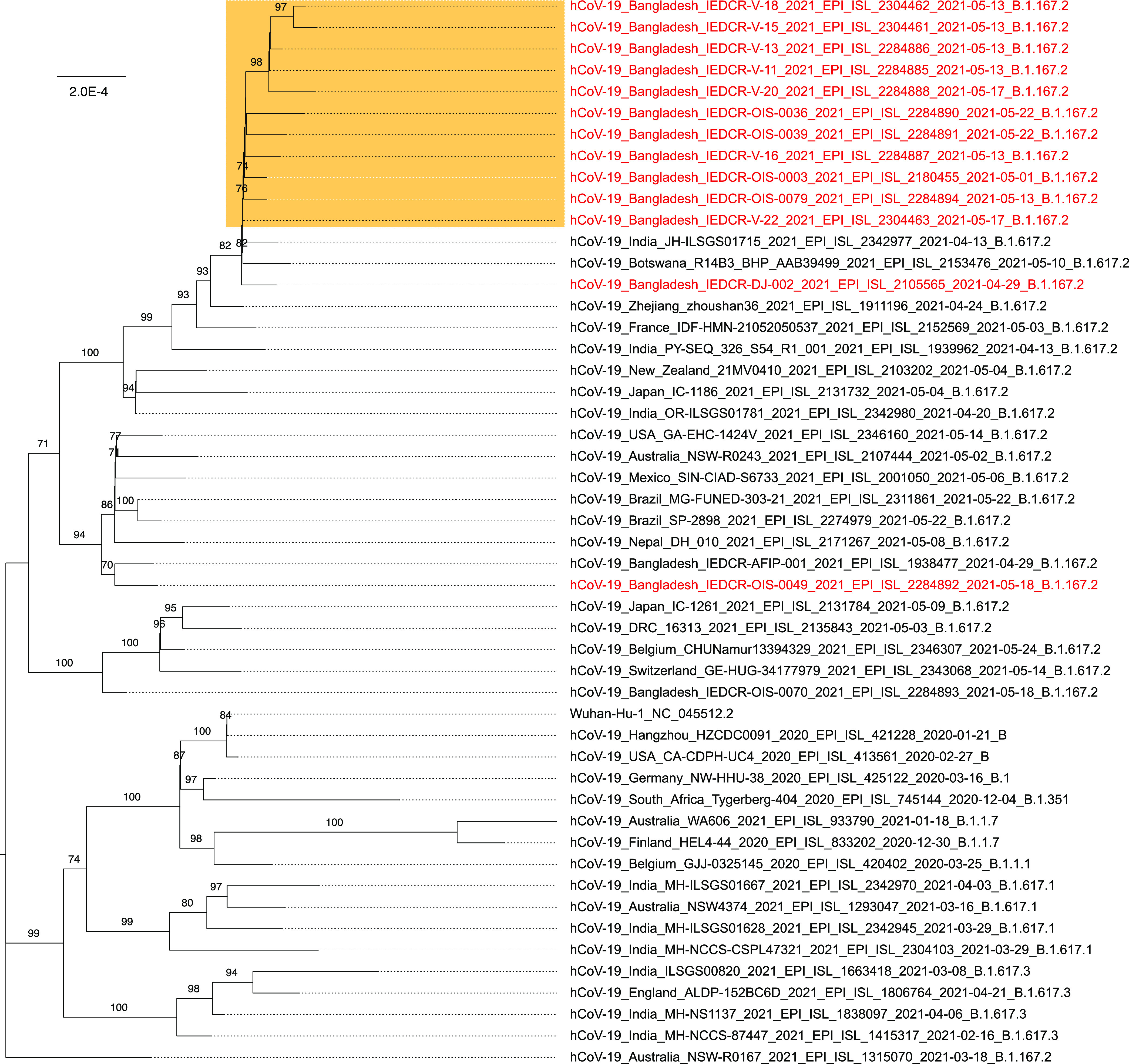
Phylogenetic analysis of coding-complete SARS-CoV-2 sublineage B.1.617.2 genomes. The orange box represents samples obtained from the bordering districts. A total of 50 viral genomes are displayed, including (i) 15 genomes from Bangladesh (highlighted in red), (ii) the SARS-CoV-2 Wuhan Hu-1 reference genome, and (iii) randomly selected genomes from GISAID representing different lineages, as well as recently isolated B.1.617.2 genomes from India. For each viral genome, the strain name, place and date of sampling, GISAID accession number, and SARS-CoV-2 lineage are indicated.

Table 1 summarizes the information for each sample. Nucleotide variations were confirmed by visual inspection using CLC Genomics Workbench v21.0 (Qiagen). Compared to the reference Wuhan Hu-1 genome, a set of 10 amino acid substitutions (shared among all 15 strains) was identified, including the five signature amino acid changes (T19R, L452R, T478K, P681R, and D950N) in the spike protein corresponding to the genetic markers of sublineage B.1.617.2. Sequences were aligned using MAFFT v7.4 (6). A phylogenetic tree (Fig. 1) was constructed with IQ-TREE v2 (7) with an ultrafast bootstrap value of 1,000 (8) and with the GTR+F+I model selected as the best-fit model by the IQ-TREE Web server (9) and was visualized using FigTree v1.4 (10). Phylogenetic analysis with Pangolin (github.com/cov-lineages/pangolin) assigned 15 samples to clade G, lineage B.1.617.2. Here, we report the early detection of SARS-CoV-2 delta variant B.1.617.2 in the Bangladeshi community, which will be useful toward mitigation of a possible third wave of COVID-19 in Bangladesh, as has been seen elsewhere.

**TABLE 1:** Data for each Bangladeshi SARS-CoV-2 sublineage B.1.617.2 isolate

Strain name	Sampling date (day/mo/yr)	Sampling location^*a*^	Patient age (yr)	Symptoms^*b*^	Vaccinated^*c*^	Recent travel to India	Genome size (bp)	Coverage (%)^*d*^	GC content (%)	GenBank accession no.	SRA accession no.
IEDCR-AFIP-001	29/4/2021	Dhaka	41	NA	No	Yes	29,769	99.55	42.4	MZ377102	SRR14800278
IEDCR-DJ-002	29/4/2021	Dinajpur	46	+	Yes	Yes	29,769	99.55	40.5	MZ377103	SRR14800279
IEDCR-OIS-0003	1/5/2021	Khulna	45	–	No	Yes	29,769	99.55	40.5	MZ377104	SRR14800272
IEDCR-V-11	13/5/2021	Chapainawabgonj	21	+	No	No	29,769	99.55	39.9	MZ377105	SRR14800271
IEDCR-V-13	13/5/2021	Chapainawabgonj	13	+	No	No	29,769	99.55	40.4	MZ377106	SRR14800270
IEDCR-V-16	13/5/2021	Chapainawabgonj	31	+	No	No	29,769	99.55	40	MZ377107	SRR14800268
IEDCR-V-15	13/5/2021	Chapainawabgonj	27	+	No	No	29,769	99.55	40.5	MZ377114	SRR14800269
IEDCR-V-18	13/5/2021	Chapainawabgonj	27	+	No	No	29,769	99.55	40.1	MZ377115	SRR14800267
IEDCR-OIS-0079	13/5/2021	Khulna	50	NA	NA	Yes	29,769	99.55	40.9	MZ377113	SRR14800273
IEDCR-V-20	17/5/2021	Chapainawabgonj	30	+	No	No	29,769	99.55	40	MZ377108	SRR14800266
IEDCR-V-22	17/5/2021	Chapainawabgonj	52	+	No	No	29,770	99.56	40.2	MZ377116	SRR14800265
IEDCR-OIS-0049	18/5/2021	Bagerhat	43	–	No	Yes	29,769	99.55	40.8	MZ377111	SRR14800275
IEDCR-OIS-0070	18/5/2021	Pirojpur	46	NA	NA	Yes	29,769	99.55	42.1	MZ377112	SRR14800274
IEDCR-OIS-0036	22/5/2021	Gaibandha	28	–	No	Yes	29,769	99.55	40.5	MZ377109	SRR14800277
IEDCR-OIS-0039	22/5/2021	Jhenaidah	49	–	No	Yes	29,772	99.56	41	MZ377110	SRR14800276

a The Khulna, Chapainawabgonj, and Jhenaidah districts are located in the western border area of Bangladesh at the Bangladesh-India border.

b +, present (fever, cough, or mild weakness); –, absent; NA, information not available.

c Yes, vaccinated with one dose of AstraZeneca COVID-19 vaccine.

d With reference to the Wuhan Hu-1 genome (GenBank accession NC_045512.2).

### Data availability.

The data from this study can be found under GISAID accession numbers EPI_ISL_1938477, EPI_ISL_2105565, EPI_ISL_2180455, EPI_ISL_2284894, EPI_ISL_2284885, EPI_ISL_2284886, EPI_ISL_2284887, EPI_ISL_2304461, EPI_ISL_2304462, EPI_ISL_2284888, EPI_ISL_2304463, EPI_ISL_2284890, EPI_ISL_2284891, EPI_ISL_2284892, and EPI_ISL_2284893. The Sequence Read Archive (SRA) and GenBank accession numbers are listed in Table 1.
